# Effect of Sporulation Conditions Following Submerged Cultivation on the Resistance of *Bacillus atrophaeus* Spores against Inactivation by H_2_O_2_

**DOI:** 10.3390/molecules25132985

**Published:** 2020-06-30

**Authors:** Philipp Stier, Ulrich Kulozik

**Affiliations:** Chair of Food and Bioprocess Engineering, Technical University of Munich, 85354 Freising, Germany; ulrich.kulozik@tum.de

**Keywords:** aseptic packaging, *Bacillus atrophaeus*, bioindicators, hydrogen peroxide, product safety, resistance, spores, sporulation, sterilization, shelf life

## Abstract

The resistance formation of spores in general and of *Bacillus atrophaeus* in particular has long been the focus of science in the bio-defense, pharmaceutical and food industries. In the food industry, it is used as a biological indicator (BI) for the evaluation of the inactivation effects of hydrogen peroxide in processing and end packaging lines’ sterilization. Defined BI resistances are critical to avoid false positive and negative tests, which are salient problems due to the variable resistance of currently available commercial BIs. Although spores for use as BIs have been produced for years, little is known about the influence of sporulation conditions on the resistance as a potential source of random variability. This study therefore examines the dependence of spore resistance on the temperature, pH and partial oxygen saturation during submerged production in a bioreactor. For this purpose, spores were produced under different sporulation conditions and their resistance, defined by the D-value, was determined using a count reduction test in tempered 35% liquid hydrogen peroxide. The statistical analysis of the test results shows a quadratic dependence of the resistance on the pH, with the highest D-values at neutral pH. The sporulation temperature has a linear influence on the resistance. The higher the temperature, the higher the D-value. However, these factors interact with each other, which means that the temperature only influences the resistance when the pH is within a certain range. The oxygen partial pressure during sporulation has no significant influence. Based on the data obtained, a model could be developed enabling the resistance of BIs to be calculated, predicted and standardized depending on the sporulation conditions. BI manufacturers could thus produce BIs with defined resistances for the validation of sterilization effects in aseptic packaging/filling lines for the reliable manufacture of shelf-stable and safe food products.

## 1. Introduction

In the food and pharmaceutical industry, sterility in production and packaging is extremely important in many instances. Pathogenic and spoilage-inducing spore formers can be particularly dangerous, since the spores, because of their high resistance, can survive pasteurization and inadequate sterilization processes and then germinate, thus contaminating the sterile product filled into nonsterile containers. As a result, they either significantly reduce the shelf life of the food or can even be harmful to consumers.

In order to prevent this, the sterility of the production facilities and all the sterilization methods within the aseptic production unit are initially validated and then regularly checked by challenge tests with so-called bioindicators (BI), since natural contaminations are variable and often not high enough to measure the target 5 log reduction effect. These BIs are test organisms, which should have a defined resistance against the respective sterilization process. Certain standard BI strains have been established, and these are routinely applied at the industrial level in validation test runs of filling machines, for instance. These BIs have a high level of resistance against certain inactivation methods using, e.g., H_2_O_2_, which covers the requirements in specific situations, e.g., in the food industry, filling neutral liquid products, or in the pharmaceutical industry. However, the resistance of commercially available BIs varies considerably.

In the case of sterilization with hydrogen peroxide, a process widely used in the food and pharmaceutical industries, *Bacillus atrophaeus* is used as a standard BI and is recognized as appropriate to cover all sorts of natural contamination. To be used as a BI, the spores of this bacterium are either immobilized on a carrier material or are used suspended in carrier liquids and exposed to the sterilization process. The performance of the sterilization can be assessed via the degree of inactivation of the BI. It is important in such applications that the resistance of the BI is defined and stable. However, in reality, depending on the manufacturer and sometimes differing batch to batch, the resistance of the BI varies. The reason for this is suspected to be that the resistance of spores is influenced by the sporulation conditions—i.e., the conditions during spore production. If the resistance differences of the BI are too large, this can lead to false positive or false negative test results when validating aseptic systems and sterilization processes.

False positive test results falsely indicate a sufficient sterilization effect when in fact this was not the case. The result then is that in regular production, nonsterile products occur at a high level. This can lead to a reduction in shelf life and, in the worst case, a risk to consumers. False negative test results, on the other hand, indicate that the sterilization was apparently not sufficient. In response to that, the sterilization conditions are tightened, which leads to over-processing, the increased use of sterilization agents, the pollution of the environment and increased costs.

The origin of variable BI resistances has not satisfactorily been addressed in research so far. The reasons are suspected to be related to the conditions during cultivation and especially during sporulation following the growth phase of the BI strain’s vegetative cells. A standardization of the production of BI of *B. atrophaeus* could solve this problem, whereby the same resistances would be obtained and uncertainties in the evaluation of challenge tests could thus be avoided.

A standardization of the production requires that the determining factors of influence which possibly affect the resistance of the spores have to be identified. Although *Bacillus* spp. and the formation of spore resistance has long been the focus of the science, bio-defense, pharmaceutical and food industries, little is known about the factors influencing its resistance against inactivation. We noticed that only a few studies in this field stem from the last few years, and some are in fact relatively old. Additionally, most studies deal with factors influencing the heat resistance of the spores. Lechowich and Ordal [1] produced spores of *B. subtilis* at 30 and 45 °C and *B. coagulans* at 30, 45 and 52 °C. They found that the spores’ heat resistance increased with an increasing sporulation temperature. Leguérinel et al. [2] and Palop et al. [3] confirm this phenomenon. However, Palop et al. [3] also state that the magnitude of the effect of the sporulation temperature is not constant—i.e., it is not well enough understood. The dependence of resistance on temperature is not linear. This contradicts Beaman and Gerhardt [4], who found that the influence of the sporulation temperature for *Bacillus* spp. spores is linear. Condon et al. [5] found that the resistance of *B. subtilis* spores shows only a partially linear dependence on the sporulation temperature and that the resistance no longer increases above a certain temperature. The pH also influences the heat resistance of spores. Nguyen Thi Minh et al. [6] produced *B. subtilis* spores at pH 6 and pH 10. They found that the spores produced at pH 10 were more resistant to heat. However, the cell growth was very poor at this pH and only a few spores could be obtained. Guizelini et al. [7] examined *Geobacillus stearothermophilus* spores for their heat resistance. They found that the resistance follows the quadratic dependence of the pH. Resistance was greatest at a sporulation pH of 8.5 and decreased towards pH 9.2 and 7.8.

Much less, however, is known about the influencing factors during sporulation on the chemical resistance of the spores. Cortezzo and Setlow [8] found that high sporulation temperatures could increase the chemical resistance of *B. subtilis* spores. However, this depends on the particular chemical. In line with Melly et al. [9], they found that the spores’ resistance to formaldehyde increases with an increasing sporulation temperature. They also obtained these effects regarding the resistance to nitrous acid and hydrogen peroxide, but the latter only for α^-^β^-^ small acid-soluble spore protein (SASP) *B. subtilis* spores. These spores are mutants that lack the genes encoding the two major α/β-type small acid-soluble spore proteins, which are relevant for DNA protection, and therefore they do not have the same protective mechanisms as wild-type spores. With regard to the resistance against ethylmethanesulphonate and methylmethanesulphonate, however, they found no influence of the sporulation temperature.

Since the mechanisms of resistance of the spores to different environmental influences are different [10], it is not possible to transfer the relationships found for heat resistance and the resistance of other chemicals to the resistance of *B. atrophaeus* wild-type spores against hydrogen peroxide, for which no such study has been reported so far. For this reason, the factors influencing the resistance of *B. atrophaeus* and their effects on resistance to hydrogen peroxide were the subject of research in this study.

The hypothesis was that the influencing factors—pH, temperature and oxygen saturation—interfere, which has not been investigated so far. In the submerged production of spores, these factors, in addition to the medium, have the greatest influence on the cells during cultivation or sporulation. For this purpose, a design of experiments was created in order to be able to make statistically significant statements and strategically examine the factors influencing the resistance. According to this experimental plan, spores of *B. atrophaeus* (ATCC 9372) were produced submerged in a bioreactor under variation in the sporulation conditions (pH, temperature and oxygen saturation), and the resistance was determined as the D-value against 35% liquid hydrogen peroxide at 25 °C. By changing the resistance of the spores depending on the sporulation conditions, conclusions can be drawn about the degree of influence and the type of dependence.

## 2. Results

In order to be able to investigate the influence of the sporulation conditions (pH, temperature, oxygen saturation in the medium) on the resistance of the spores, a model had to be developed with which it is possible to make statistically significant statements about the influences on the resistance by varying the sporulation conditions. For this purpose, a full factorial experimental design (Design of Experiments, DoE) was developed. With factorial test plans, the influences of several factors on one (or more) target variables can be examined. The influencing variables are measurable variables, in this case the temperature, the pH and the oxygen saturation of the medium. With 2–3 influencing factors, a two-stage test plan is usually used—i.e., each influencing variable is examined at two levels (minimum and maximum value).

Therefore, these minimum and maximum values must first be determined. This step of modeling is extremely critical in microbiology. If these extremes are too far from the growth optimum of the bacterium, spores could possibly no longer be formed or growth inhibition could occur and falsify the result. However, if these values are too close to each other—i.e., too close to the growth optimum of the bacterium—the measured data may be very similar to the target (D-value), so that no significant dependencies can be determined. In addition, when building the model, it must be taken into account that not only linear dependencies (1st order effects) of the influencing variables on the target variable are possible, but also non-linear dependencies (2nd order effects). If a factor was only examined in two stages, there is a risk of missing a quadratic relationship. To exclude this, so-called “center points” are included in the model, which allows us to assess whether the resistance of the spores has a linear or non-linear dependence on the influencing factors. The center points are to be understood as mean settings—i.e., as the mean between the respective extremes of the influencing variable.

For the modeling, preliminary tests were therefore carried out in the bioreactor to determine the extremes. The extremes were set to pH 6.0 and 8.5, with a center point of 7.25; for temperature, they were set to 28 °C and 40 °C, with a center point of 34 °C; and for the minimum oxygen saturation, they were set to 10%, 60% and 35%. An experimental plan was determined in accordance with this range of values, and spores were produced in a bioreactor under the specified sporulation conditions. Therefore, the cells were enriched in a culture medium and inoculated with an optical density (OD_600_) of 1 into the bioreactor. The sporulation medium provides the cells with enough nutrients for exponential growth for about 4 h. Then, the sporulation of the cells began due to starvation—i.e., when the carbohydrate source was used up—and after 48 h, the spores were harvested. At this point, it is important to allow the spores to fully mature, which is why it is essential to avoid harvesting the spores too early. The temperature and pH were kept constant over the entire time in the bioreactor. The oxygen saturation in the medium decreased from saturation (100%) to the set value with the beginning of the exponential growth phase due to cell metabolism. The oxygen level was kept at the set value during the growth phase until the beginning of sporulation and then increased again, because no oxygen was consumed anymore. After the produced spores were harvested and purified, the resistance of the spores was determined as the D-value against 35% liquid hydrogen peroxide at 25 °C.

When the D-values of the test series were determined, a model alignment was carried out. In this alignment, the influences of the variables on the target (D-value) were analyzed and the model adjusted accordingly. In this case, it was found that the dependencies are not purely linear, but that the pH has a quadratic influence on the D-value. Table 1 shows the full factorial DoE with the sporulation conditions and the resulting measured and calculated D-values. It can be seen that the measured D-values of the test series match the calculated D-values of the adapted model very well. Experiment No. 9 is an exception, which is why it is not included in the calculations. The spores of this experiment had an atypically dark color after harvesting and did not show the light refraction typical for spores under a light microscope. This is most likely an error during sporulation in the bioreactor. It is therefore not surprising that the D-value differs from experiment No. 2, which was carried out under the same conditions.

**Figure 1:**
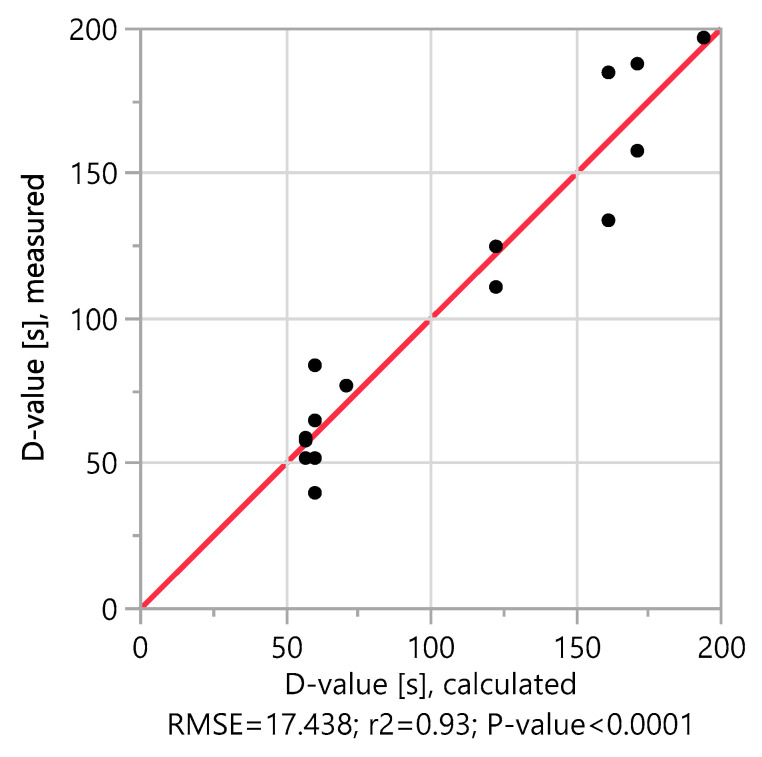
Observed values versus prediction. The diagram shows the actual measured D-values compared to the predicted calculated D-values. For a good approximation, the points on the scatter diagram must move to the red line. The points are all very close to the line, which means that the model maps the dependencies very well. Another indicator of model accuracy is the r^2^ value. This measures the percentage of variability in the D-value that is explained by the model. A value closer to 1 means that the model gives a good forecast (in this case, 0.93).

The measured D-values of the 16-test series are between 40 and 197 s and fit very well with the calculated D-values of the model. This is confirmed by plotting the measured D-values against the calculated D-values in Figure 1, with an r^2^ value of 0.93. The model alignment also shows that the oxygen saturation in the medium has no significant influence on the D-value. The oxygen saturation is therefore not taken into account in further model calculations.

Based on the D-values, the model yields an equation (Equation (1)) by which it is possible to predict all the D-values within the model range—i.e., within the extremes of the influencing factors temperature and pH:

(1) $$\begin{array}{c} \mathrm{D-value}\;\left[s\right]=171.13781504+\left(-28.78900087\right)*\left(\frac{\left(\mathrm{pH}-7.25\right)}{1.25}\right)+21.772689936\\ \;*\left(\frac{\left(\mathrm{Temp}.\;\left[{}^{\circ}C\right]-34\right)}{6}\right)+\left(\frac{\left(\mathrm{pH}-7.25\right)}{1.25}\right)\\ \;*\left(\left(\frac{\left(\mathrm{Temp}.\;\left[{}^{\circ}C\right]-34\right)}{6}\right)*\left(-23.37179094\right)\right)+\left(\frac{\left(\mathrm{pH}-7.25\right)}{1.25}\right)\\ \;*\left(\left(\frac{\left(\mathrm{pH}-7.25\right)}{1.25}\right)*\left(-83.90606869\right)\right) \end{array}$$

**Figure 2:**
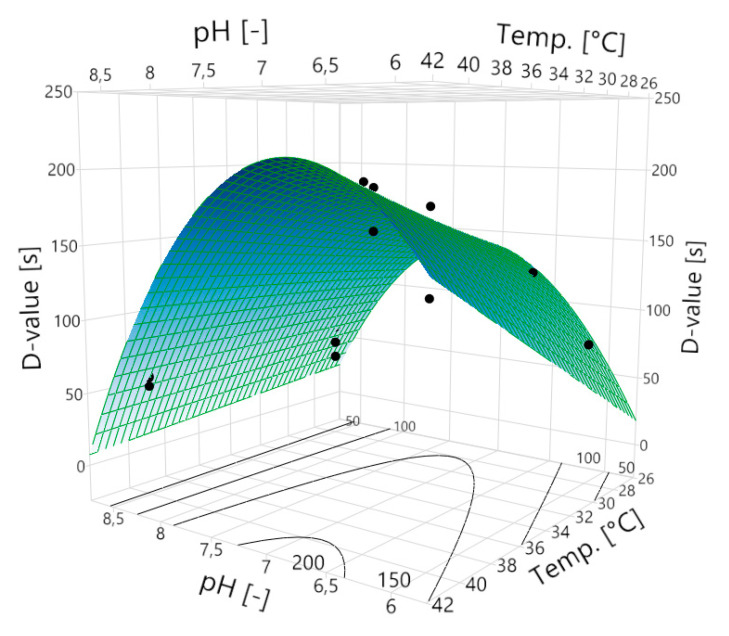
Dependence of the D-value on the temperature and pH. The surface plot shows the expected D-values for the respective sporulation conditions. The black data points are the actually measured D-values on which the model is based. Some of the values are hidden by the surface. The D-value is greatest at a high temperature of 40 °C and a pH of 6.9. If the temperature drops or the pH changes to acidic or basic, the D-value also decreases.

**Figure 3:**
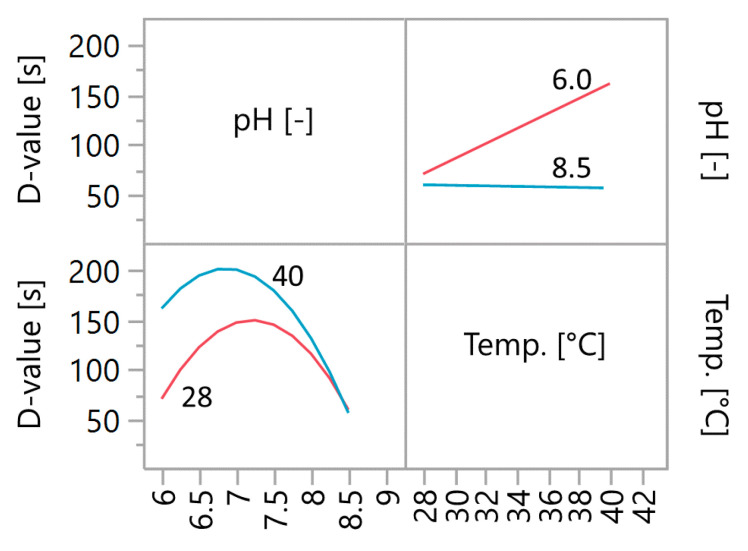
Interaction profiles of pH and temperature. The influence of temperature on the D-value increases with decreasing pH. The pH value at which the greatest D-value is expected shifts from 7.2 to 6.9 when the temperature rises from 28 to 40 °C.

Using this equation, a three-dimensional prediction model of the D-value on the temperature and the pH can be established (Figure 2):

The highest D-value of 200 s is obtained at pH 6.9 and 40 °C. The expected D-value drops towards lower and higher pH values. Depending on the selected pH value, the temperature has a different effect on the D-value. At a low pH such as 6.0, the D-value shows a very strong linear dependence on the temperature, which means that with increasing temperature, the D-value also increases. At the pH of 8.5, increasing the temperature does not increase the D-value. This interaction of the two influencing variables becomes obvious in Figure 3.

With increasing pH, the influence of the temperature decreases to such an extent that from pH 7.8 to 8.5, the temperature no longer has a significant influence on the D-value. Thus, at pH 8.0 D-values from ~115 s to ~131 s are predicted at temperatures between 28 and 40 °C, but it can no longer be said that there is a significant difference. If the pH is further increased to 8.5, the D-value in the entire temperature range is predicted to be ~59 s. There is no longer a difference in the D-value between 28 °C and 40 °C. This makes the pH the only variable that influences the resistance of the spores. The greatest effect of the temperature on the D-value is at the slightly acidic pH of 6.0. Here, D-values from ~70 s to ~160 s are to be expected (increasing from 28 °C to 40 °C). Towards pH 7.0, the influence of temperature on the D-value softens but is still significant. Thus, across the range of investigated temperatures, D-values of ~150 s to ~200 s are predicted at pH 7.0.

In addition, the influence of the pH on the D-value changes as well, depending on the temperature. At 28 °C, the highest D-value is expected at a pH of 7.2. The D-value decreases towards lower and higher pH values. At a temperature of 40 °C, the highest D-value is expected at a pH of 6.9. This means that the pH at which the greatest D-value is expected shifts with rising temperature. Overall, the prediction model shows D-values in the range of ~59 s to ~200 s.

## 3. Discussion

In this study, it could be shown that the resistance of submerged produced spores to hydrogen peroxide depends largely on the pH and temperature. The pH has a quadratic influence on the resistance, which means that there is an optimum at which the resistance reaches its maximum. If the pH changes further to acidic or basic, the resistance of the spores also decreases. The resistance, however, shows a linear dependence on temperature. With increasing temperature, the resistance of the spores to hydrogen peroxide also increases. It should be noted that there is an interaction between pH and temperature which means that the factors influence each other. This has a significant impact on the degree of the effect of temperature on the resistance of the spores. The influence of temperature is strongest in the neutral and acidic pH range. When the pH becomes more alkaline, the effect of the temperature is reduced until the temperature finally has no influence on the resistance. The oxygen saturation of the medium has no significant influence on the resistance of the spores.

It should be noted that the results apply to *B. atrophaeus* only in the examined area of the DoE with the media and methods used. It is generally known that the sporulation medium also has an influence on the resistance of bacterial spores. However, it can be assumed that the dependencies found qualitatively also apply to other media in the submerged production of *Bacillus* spores, possibly even their quantity. Whether and to what extent there is an interaction of the temperature and the pH with the medium or its composition (main ingredients, concentration and presence of manganese, iron, zinc, calcium, etc.) and whether this affects the resistance of the spores to hydrogen peroxide was not examined in this study.

The effect of the sporulation temperature on the resistance of *Bacillus* spores has already been investigated in many publications [1,2,3,4,5,9,11,12,13,14,15,16]. The spore structure was found to be decisive for their resistance. It is believed that treating the spores with hydrogen peroxide primarily damages the germination apparatus and the inner membrane. Core proteins may also be affected by the damage [17]. The spores are probably protected by superoxide dismutases in the coat and catalase activity, which are also associated with the outer layers of the spore [18,19]. Furthermore, polycyclic terpenoids (sporulenes) contribute to the resistance of the spores to hydrogen peroxide [20]. α/β-type small acid-soluble spore proteins (SASPs) protect the DNA in the spore core [17]. The low water content of the spores also contributes to their resistance and is influenced by the sporulation temperature [21]. The higher the sporulation temperature, the lower the water content of the spores will be. The result is that of an increase in the resistance of the spores to heat [4,9]. Above a certain temperature, however, the resistance decreases again, so it is not linear—e.g., without limit [11]. An increase in resistance to chemicals such as formaldehyde could also be shown by increasing the sporulation temperature [8,9] and can be confirmed in this study in the case of hydrogen peroxide depending on the pH value during sporulation. A reduction in the water content presumably slows down chemical reactions and thus protects the spores [17].

It is not mechanistically clear what influence the sporulation pH has on the resistance of the spores, but it is known that the pH influences the structure of the spores during sporulation. Depending on the pH, the surface hydrophobicity [22] or the size and shape of spores can change [23]. It is not known, however, whether and how this affects the resistance of the spores to liquid hydrogen peroxide.

The spores have the greatest resistance when the pH is in a range that corresponds approximately to the optimum growth of the spore formers. This is in line with the results of Guizelini et al. [7], who found a quadratic influence of the pH on the resistance of submerged produced spores of *Geobacillus stearothermophilus*, the highest resistance being at a sporulation pH of 8.5 and decreasing at 7.8 and 9.2. A sub-optimal pH value may have a negative influence on the bacteria even in a vegetative stage and/or when sporulation and spore maturation are inhibited, which means that the resistance of the spores cannot be fully developed.

The interaction of pH and temperature during sporulation and their mutual influence on resistance is striking. Apparently, the temperature has only a minor influence, since its effect on the resistance only comes into play in a certain pH range. From this, it can be concluded that the influence of the pH on the resistance interferes more fundamentally with the structural effects influencing the resistance of the spores than the temperature does. A reduction in the water content of the spores can be neglected if a sub-optimal pH value has a fundamental influence on the structure of the spores.

## 4. Materials and Methods

### 4.1. Strain

Spores of *Bacillus atrophaeus* (ATCC 9372/DSM 675) with a concentration of 10^7^ colony forming units (CFU)/mL, suspended in 70% ethanol were stored at −80 °C. The storage conditions ensured that the properties of the spores did not change over the experimental period. The spores were aliquoted at 1000 µL each. This enabled the spores to be taken without the need to thaw the remaining spore suspension.

### 4.2. Bioreactor

A 2 L bioreactor (Biostat A, Sartorius AG, Goettingen, Germany) was used for the experiments. It enabled the fully automatic control of the pH value, the temperature and the oxygen saturation. The temperature and pH were kept constant at the set value during the spore production. The pH was adjusted with 0.5 M of NaCl and 0.5 M of NaOH. The set oxygen saturation corresponded to the minimum permissible oxygen saturation. If this value was undershot, the gas intake automatically increased first and then, if necessary, the stirring speed. These adjustments kept the oxygen saturation at the set value. Korasilon FG 30 (Kurt Obermeier GmbH & Co. KG, Bad-Berleburg, Germany) served as an anti-foaming agent. The anti-foaming agent was added automatically when needed.

### 4.3. Media

Terrific Broth (casein peptone 12 g/L (for microbiology, Gerbu Biotechnik GmbH, Heidelberg, Germany), yeast extract 24 g/L (for microbiology, Merck KGaA, Darmstadt, Germany), K_2_HPO_4_ 9.4 g/L (ACS reagent, ≥98%, Merck KGaA), KH_2_PO_4_ 2.2 g/L (ACS reagent, ≥99%, Merck KGaA), glycerol 8 g/L (Rotipuran ≥99.5%, Carl Roth GmbH & Co. KG, Karlsruhe, Germany)) was used as the pre-culture medium. This medium enabled a rapid increase in the cell density of *B. atrophaeus* without showing any effect on the vitality of the cells.

Modified Difco Sporulation Medium (modified from [24]; casein peptone 5 g/L (for microbiology, Gerbu Biotechnik GmbH), beef extract 3 g/L (for cell biology, Gerbu Biotechnik GmbH), KCl 3.5 g/L (ACS reagent, 99.0–100.5%, Merck KGaA), MgSO_4_ · 7H_2_O 0.25 g/L (ACS ≥99%, Carl Roth GmbH & Co. KG), 30% glucose 10 mL/L (for biochemistry, Reag. Ph Eur., 97.5–102.0%, Merck KGaA), 1 M Ca(NO_3_)_2_ · 4H_2_O 1 mL/L (ACS reagent, 99%, Merck KGaA), 10 mM MnCl_2_ · 4H_2_O 1 mL/L (ACS reagent, ≥98%, Merck KGaA), 1 mM FeSO_4_ · 7H_2_O 1 mL/L (ACS reagent, ≥99%, Merck KGaA)) was used for sporulation in the bioreactor. In this medium, *B. atrophaeus* showed a high sporulation rate with a very low cell mortality.

### 4.4. Pre-Culture

An aliquot of 1000 µL of the stored spore suspension was inoculated in 400 mL of Terrific Broth in a 1 L baffled shaking flask. Incubation took place at 30 °C, 135 rpm for 17 h. The cell suspension was then pelleted by centrifugation at 4000× *g*, 4 °C for 10 min. The supernatant was discarded and the cell pellet was resuspended with 25 mL of ultrapure water.

### 4.5. Spore Production in the Bioreactor

The bioreactor was inoculated with an OD_600_ of 1 with the cells of the pre-culture. Incubation took place for 48 h. The suspension was then harvested and checked for the presence of spores by light microscopy.

### 4.6. Spore Purification

The harvested spore suspension was purified prior to the determination of resistance to remove metabolites and residual media. For this purpose, the spore suspension was washed three to five times with dist. water (4000× *g*, 10 min, 4 °C) and suspended. Subsequently, the suspension was shaken overnight at 480 rpm in order to dissolve the agglomerates of remaining cell debris and to release spores. This was followed by three more washing steps and subsequent centrifugation for 90 min at 6000× *g* and 4 °C. This step caused the formation of a very solid three-phase pellet. The upper two phases had to be removed. They included cell debris and maternal cells with endospores. The lower phase contained free spores. For cleaning, the supernatant was discarded and fresh dist. water was added carefully to the pellet. The pellet was now mixed rigorously. Since the lower spore phase was very firm due to centrifugation, only the upper phases (slimy consistency) dissolved. By replacing the supernatant, this step was repeated until the upper phases were removed. Finally, the remaining pellet could be dissolved with dist. water and stored at 4 °C.

### 4.7. Determination of the D-Value

In order to be able to compare the resistance of the spores due to the variation in the sporulation conditions, the D-value had to be determined. For this purpose, 100 µL of the purified spore suspension was exposed to 9.9 mL of a liquid solution of 35% hydrogen peroxide (Oxteril 350 Spray, Food Grade, Evonik Industries AG, Essen, Germany) at 25 °C and stirred at 480 rpm. In the course of the inactivation reaction, 100 µL of the samples were taken in defined time intervals and the reaction was stopped in a 1:10 dilution of dist. water with catalase (Catalase from *Micrococcus lysodeiktikus*, solution, activity 65,000−150,000 U/mL, Merck KGaA, Darmstadt, Germany). Then, appropriate dilutions were plated on a Plate Count Agar and incubated for 48 h at 30 °C. The decrease in CFU/mL over the course of treatment with hydrogen peroxide could be used to visualize the inactivation course and determine the D-value. The D-value is the time required, at a given set of conditions, to achieve a 1-log reduction.

## 5. Conclusions

In this study, it could be shown that the resistance of the submerged produced spores of *B. atrophaeus* is dependent on the pH as well as the incubation temperature. The key finding is that these influencing factors interact with each other, and therefore the temperature only influences the resistance in a certain pH range and is subordinate to the pH as the main influencing factor. These results help us to understand the effects of sporulation conditions on the variability of resistance of BIs better. It could be shown how important the understanding of the formation of resistance of spores is in order to reduce or prevent resistance fluctuations in BIs. In terms of scientific progress, this outcome shows that the formation of resistance by spores is influenced by many factors that mesh with one another, can influence one another, or are dependent on one another. Therefore, such influencing factors should no longer be examined individually in the future or only with the greatest caution. Regarding practical applications, it can be stated that making use of our results in the production of commercial BIs could be a significant step forward towards more reliable results in sterilization test runs—e.g., as part of the commissioning of newly installed production or filling lines. However, another practical implication would be that all stakeholders in this context would have to agree on implementing these findings in producing BIs with standardized resistance. Additionally, it would be required to agree on the target resistance level. Standardizing the production of BIs based on these findings on the formation of spore resistance could help to increase the product shelf life and safety in the food and pharmaceutical industries and to counteract uncertainties in the validation of sterilization systems and methods. Further work in this field is required to address other factors such as the sporulation medium composition in relation to sporulation-influencing components (e.g., manganese, zinc, iron and calcium) and to transfer this kind of approach also to other established BIs—e.g., those of *G. stearothermophilus* applied as BI in pharmaceutical applications.

## Figures and Tables

**Table 1:** Experimental plan and results of the full factorial Design of Experiments (DoE) to investigate the influence of sporulation conditions (pH, temperature and oxygen saturation) on the resistance (D-value) of the spores of *B. atrophaeus* in the bioreactor.

No.	pH [-]	Temp. [°C]	pO_2_ [%]	D-Value, Measured [s]	D-Value, Calculated [s]
1	8.5	28	10	40	60.04
2	6.0	28	60	77	70.88
3	8.5	28	10	65	60.04
4	6.0	40	10	134	161.17
5	6.0	40	60	185	161.17
6	8.5	28	60	84	60.04
7	7.25	34	35	158	171.14
8	7.25	34	35	188	171.14
9 *	6.0	28	60	171	70.88
10	8.5	40	60	58	56.84
11	8.5	40	10	59	56.84
12	6.5	28	10	125	122.41
13	8.5	40	10	52	56.84
14	8.5	28	60	52	60.04
15	6.5	40	60	197	194.00
16	6.5	28	10	111	122.41

* The measured D-value of the 9th test run was rated as an outlier and was therefore not taken into account in the evaluation.
